# In memory of Michael R. Detty, PhD

**DOI:** 10.7150/ntno.63716

**Published:** 2022-01-01

**Authors:** David Watson, Stefan Harmsen

**Affiliations:** 1 Department of Chemistry, University at Buffalo, Buffalo, NY, 14260, USA; 2 Department of Radiology, University of Pennsylvania, Philadelphia, PA, 19104, USA

On 1 July 2020, the chemistry community sadly lost Michael R. Detty, a professor and former chair in the Department of Chemistry of the University at Buffalo. Testimonials from David Watson and colleagues from University at Buffalo, and Stefan Harmsen from University of Pennsylvania, highlight Professor Detty's accomplishments as researcher, inventor, teacher, mentor, collaborator, and colleague, and his lasting impact on dye chemistry.

## Making an impact at the University at Buffalo

Mike Detty was a native of Ohio. He obtained his bachelor's degree in chemistry from Bowling Green State University and his PhD in organic chemistry from The Ohio State University. After completing his PhD, Mike spent 17 years as a research scientist at the Eastman Kodak Company in Rochester, NY, where he developed a range of dye chromophores, focusing particularly on IR-absorbing dyes. His work at Kodak yielded 26 patents and 62 peer-reviewed articles.

In 1995, Mike joined the faculty of the University at Buffalo (UB), initially in the Department of Medicinal Chemistry and then in the Department of Chemistry following the merger of the two departments. Mike made lasting contributions in research, teaching, and service, and he truly relished the opportunity to contribute in all three pillars of academia.

Mike expanded his prolific research career at UB, applying synthetic organic chemistry and the design of dyes and other compounds to wide-ranging problems in medicinal chemistry, materials science, catalysis, solar energy conversion, biofouling, and biomedical imaging. Mike's research on heavy chalcogen dyes is world-renowned. A hallmark of Detty group research was its collaborative nature; he and his group engaged in many successful collaborations with researchers at UB and worldwide. While at UB, Mike authored over 130 peer-reviewed publications, obtained seven more U.S. patents, and received UB's Visionary Inventor Award and the Jacob F. Schoellkopf Medal from the Western New York section of the American Chemical Society.

Mike was deeply committed to training and mentoring young scientists, from undergraduate and graduate students in the classroom and laboratory to young faculty colleagues beginning their independent careers. Mike graduated twenty-plus PhD students and mentored countless undergraduate researchers, and additionally was a skilled and dedicated classroom teacher. Mike's superior teaching and mentoring was recognized with the SUNY Chancellor's Award for Excellence in Teaching, the Milton Plesur Award from UB's Undergraduate Student Association, and the Meyerson Mentoring Award for Distinguished Undergraduate Teaching and Mentoring.

Mike was also an accomplished administrator. He was chair of the Department of Chemistry for four years, and he then served for two years as Interim Director of UB's Center for the Arts. During the latter role, Mike merged his administrative prowess with his love of music and the performing arts, and he found yet another way to move our university forward.

Testimonials from faculty and former students echo these themes:

*From Dr. Kellie Gast, former PhD student with Prof. Detty and currently Assistant Professor of Chemistry at St. Bonaventure University:* “Dr. Detty was one of the main reasons I joined the UB Chem department for graduate school. He helped me through an REU in the summer of 2010 and welcomed me back with open arms in the summer of 2012. I had dreams of doing a project with cancer research and Dr. Detty made that happen for me. We were fortunate enough to get experience with synthesis and then testing at Roswell Park. Dr. Detty is also one of the main reasons I went into teaching. He truly was a great teacher and mentor. He knew when to give us some tough love, but then would always follow it up by bringing in some bagels for us to have breakfast on an early morning in the lab.”

*From Dr. Konstantinos Plakas, former PhD student with Prof. Detty and currently a postdoctoral research fellow at the University of Pennsylvania:* “Dr. Detty was a supportive mentor, colleague, and friend. He was supportive in guiding me in my development as a scientist while also demonstrating patience as he granted me the freedom to formulate my own questions and solutions. His expertise in the field of dye chemistry and his genuine passion for problem-solving is evident through his myriad of internal and external collaborations and long-lasting professional relationships. He was also incredibly supportive of undergraduate students, often ensuring that each graduate student had a mentee to work with.”

*From Prof. Sherry Chemler:* “Mike was an outstanding role model and mentor. The high standards he had for his students and colleagues was modeled by the high standards he had for himself. He was a strong advocate for merit-based professional advancement and was ahead of his time in tackling issues of equity and inclusion. He was generous in sharing his scientific expertise, which ranged from organic to medicinal to physical chemistry. He was also generous in sharing the wisdom gained through his personal life experience. Mike's strong moral compass and giving nature was evident throughout his personal and professional life and he is missed.”

*From Prof. Steve Diver:* “It was a pleasure serving on the chemistry faculty with Mike Detty for the past two decades. For brevity, I will overlook the distinction Mike achieved in his research and instead reflect on a few personal qualities that I came to respect over the years. These positive attributes uplifted others and enhanced teaching and research in the department.

“First, Mike communicated effectively with students of all levels. We give brief presentations each year to pitch our research projects to new graduate students; it amazed me to see how persuasive Mike was and how well he communicated the impact of his work. His effective communication may explain his second quality, his love of teaching. Maybe all those years at Kodak saw his natural talent for teaching repressed, and it came free when he started in academics at UB! Mike enjoyed teaching medicinal and organic chemistry and remarkably, it seemed to come easy to him. Because of their importance in pharmaceuticals, Mike was always chomping at the bit to teach students about heterocyclic chemistry! At the drop of a hat, he could teach a class on this subject. He covered for me many times and taught heterocycles recently in my organic class. Third, Mike relentlessly praised and promoted his students. He effectively conveyed his respect for them as individuals and was able to both praise and articulate their accomplishments. This support may help explain many of the successful careers of Detty group alumni. Last, Mike championed our junior colleagues, the new assistant professors. I think he valued their new ideas, the fresh research projects and maybe he liked the opportunity to share some of his learned wisdom. This had a positive impact on our faculty development.

“This glimpse of Mike shows what an influential faculty member he was. Seeing his example helped me re-affirm my commitment to serve my students and to strive for teaching excellence. His retirement was too short and he is missed by faculty and students here at UB.”

Mike cherished his role as a member of UB's faculty. He positively impacted the lives of countless students and colleagues, and he made myriad contributions in research, teaching, mentoring, technology transfer, and administration. Mike left a lasting impression at UB, and the impact of his work will live on. He is sorely missed by faculty, staff, and students.

-David Watson

## Inspiring Research

Mike was not only a great mentor, educator and friend, but also an outstanding dye chemist whose work on chalcogenopyrylium-based dyes for the application of photodynamic therapy (PDT), among others, was ahead of its time. It therefore comes as no surprise that Mike published on a regular basis in the *Journal of Medicinal Chemistry*, *Journal of Organic Chemistry (JOC),* and the* Journal of the American Chemical Society (JACS)*, and that his work has featured in many reviews related to dye chemistry and applications; one of which was the seminal review “Near-infrared absorbing dyes” by J. Fabian *et al.* in *Chemical Reviews* (1992) 1. It was this review that led to my collaboration with Mike that ultimately resulted in the development of chalcogenopyrylium dyes as Raman reporters for surface-enhanced Raman scattering (SERS) nanoprobes that produced unparalleled signal intensities 2 - an achievement that was heralded as a key development in the SERS research field by Laing *et al.* in their *Nature Reviews Chemistry* article (2017) 3.

Mike's knowledge, experience, and creativity was instrumental in the design and optimization of the chalcogenopyrylium-based Raman reporters, which due to their molecular structures produced unique Raman fingerprints. In SERS, the Raman signal enhancement is dependent on the interaction of the Raman reporter with the noble metal nanoparticle surface. Therefore, Mike designed resonant chalcogenopyrylium dyes with thiophene functional groups that were not only part of the chromophore, but also functioned as anchors to attach these dyes to the noble metal nanoparticle surface. Using these novel dyes as resonant Raman reporters in Moritz Kircher's Lab at Memorial Sloan Kettering Cancer Center, we produced SERRS nanoparticles with attomolar sensitivity 2. Together with researchers at the University of Strathclyde, Mike demonstrated how the contact angle of the Raman reporter on the gold nanoparticle surface dictated its Raman signature opening up a whole new realm of Raman-based sensing applications 4.

When I moved to Stanford, Mike and his then-graduate student Kosta Plakas visited me and I showed them around. During the campus tour, Mike talked about his career and how important his family was for him. He left early after the dinner and trusted the “younger generation” to come up with the terms of the continued collaboration. This was the last time I saw Mike in person. Later that year, he called me about the results of our study on triple-bond containing Raman reporters, which are featured in this *Special Issue*. He was really excited and had many plans on how to build on these results with better dye designs and modeling. He also shared with me that he was having health issues and had undergone extensive surgery. He sounded very optimistic about the future, but unfortunately this would be the last time I talked to him.

I am honored to have been in the opportunity to work with Mike and learn from him professionally and personally. His contributions have elevated the scope and impact of not only our research, but also that of others and the field as a whole.

Dr D., you will be missed!

-Stefan Harmsen
